# Identification of Falsified Chloroquine Tablets in Africa at the Time of the COVID-19 Pandemic

**DOI:** 10.4269/ajtmh.20-0363

**Published:** 2020-05-12

**Authors:** Gesa Gnegel, Cathrin Hauk, Richard Neci, Georges Mutombo, Fidelis Nyaah, Dorothee Wistuba, Christine Häfele-Abah, Lutz Heide

**Affiliations:** 1 Pharmaceutical Institute, Eberhard Karls University Tuebingen, Tuebingen, Germany;; 2 German Institute for Medical Mission (Difaem), Tuebingen, Germany;; 3 Ecumenical Pharmaceutical Network, Nairobi, Kenya;; 4 Le Dépôt Central Médico-Pharmaceutique de La 8e CEPAC (DCMP), Bukavu, Democratic Republic of Congo;; 5 Presbyterian Church in Cameroon (PCC), Central Pharmacy, Limbe, Cameroon;; 6 Institute of Organic Chemistry, Eberhard Karls University Tuebingen, Tuebingen, Germany

## Abstract

Reports that chloroquine and hydroxychloroquine may be effective against COVID-19 have received worldwide attention, increasing the risk of the introduction of falsified versions of these medicines. Five different types of falsified chloroquine tablets were discovered between March 31, 2020 and April 4, 2020, in Cameroon and the Democratic Republic of Congo by locally conducted thin layer chromatographic analysis. Subsequent investigation by liquid chromatography and mass spectrometry in Germany proved the absence of detectable amounts of chloroquine and the presence of undeclared active pharmaceutical ingredients, that is, paracetamol and metronidazole, in four of the samples. The fifth sample contained chloroquine, but only 22% of the declared amount. Such products represent a serious risk to patients. Their occurrence exemplifies that once medicines or vaccines against COVID-19 may be developed, falsified products will enter the market immediately, especially in low- and middle-income countries (LMICs). Timely preparations for the detection of such products are required, including the establishment of appropriate screening technologies in LMICs.

In February 2020 and March 2020, reports that chloroquine (CQ) and hydroxychloroquine (HCQ) may be effective against COVID-19^1–4^ received massive political and media attention worldwide, despite limited evidence.^5,6^ Concerns have been raised that the premature off-label use of CQ and HCQ in COVID-19 may result in shortages of these medicines in their established, approved indications (i.e., against autoimmune diseases and, in case of CQ, *Plasmodium vivax* malaria).^7,8^ The demand for CQ and HCQ quickly outstripped the supply, exacerbating the risk of falsified medicines entering the market.^8^ We here report the recent occurrence of falsified CQ, detected in Cameroon and the Democratic Republic (DR) of Congo.

The Ecumenical Pharmaceutical Network (EPN), among other tasks, monitors medicine quality using the Global Pharma Health Fund (GPHF) Minilab,^9^ a screening methodology based on thin layer chromatography (TLC) which is easy to conduct in resource-limited environments.^10^ In March 2020, local member organizations of the EPN reported that both in private pharmacies and in informal markets, several types of falsified CQ tablets were appearing which, in local GPHF Minilab analysis,^11^ were found not to contain CQ. Through the German Institute for Medical Mission (Difaem), the member organization of EPN which coordinates the Minilab network, the WHO Rapid Alert System, was informed, and the WHO published an international Medical Product Alert about falsified CQ tablets.^12^

In the following days, further falsified CQ samples were identified in Cameroon. Five samples were forwarded by commercial courier from Cameroon and the DR Congo to Tuebingen University, Germany. They are depicted in Figure 1, together with photos of their TLC analysis, according to the GPHF Minilab procedure.^11^ Details of the samples are listed in Table 1.

**Figure 1.**
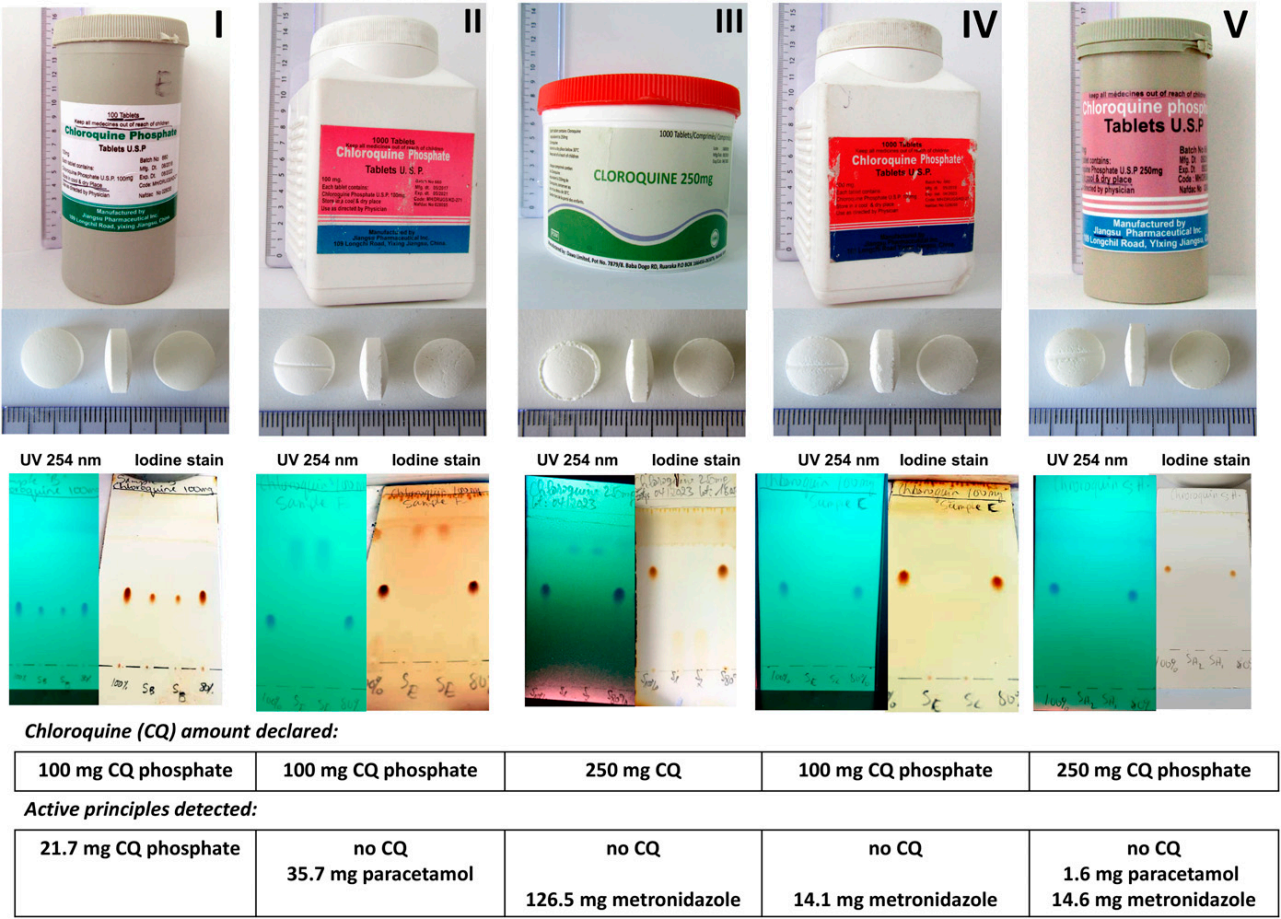
Falsified samples of chloroquine (CQ) tablets identified in Cameroon and the Democratic Republic Congo, and their thin layer chromatographic (TLC) analysis^11^; see Supplemental Information for details of the analytical procedure. Each TLC plate shows two spots of the respective sample in the middle and two spots of authentic CQ (corresponding to 100% and 80% of the declared amount of the sample) on the left and the right, respectively. Thin layer chromatography plates were photographed in Cameroon and the Democratic Republic Congo with locally available equipment; therefore, the angle of photography is not uniform. The active principles listed at the bottom were identified by high-performance liquid chromatography according to the U.S. Pharmacopeiea and by liquid chromatography–high-resolution mass spectrometry analysis (see text). The CQ amount in sample I was calculated as CQ phosphate; the identity of the counterion (phosphate or sulfate) was not determined. (Photos: packaging,^©^ G. G., C. H., and L. H.; TLC analysis,^©^ F. N. and G. M.)

**Figure 2.**
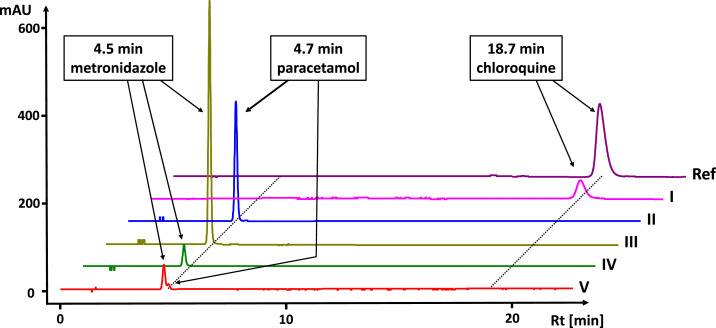
High-performance liquid chromatography analysis of falsified samples of chloroquine (CQ) tablets. Analysis was carried out according to the U.S. Pharmacopeia^13^; see Supplementary Information for details of the analytical procedure. Ref = CQ authentic reference substance; I, II, III, IV, and V = falsified samples of CQ tablets (see Figure 1, Table 1).

**Table 1:** Falsified samples of chloroquine tablets identified in Cameroon and the DR Congo

Sample code (Figures 1 and 2)	I	II	III	IV	V
Stated product name	Chloroquine phosphate tablets U.S.P	Chloroquine phosphate tablets U.S.P.	Cloroquine [*sic*] 250 mg	Chloroquine phosphate tablets U.S.P.	Chloroquine phosphate tablets U.S.P
Stated strength	100 mg	100 mg	250 mg	100 mg	250 mg
Stated manufacturer	Jiangsu Pharmaceutical Inc., China	Jiangsu Pharmaceutical Inc., China	Dawa Limited, Kenya	Jiangsu Pharmaceutical Inc., China	Jlangsu [*sic*] Pharmaceutical Inc., China
Batch number, mfg date, exp date	660, August 2018, August 2022	660, May 2017, May 2021	1605059, May 2019, April 2023	660, May 2019, April 2023	660, September 2018, September 2022
Found in	Limbe, Cameroon	Douala, Cameroon	Bukavu, DR Congo	Douala, Cameroon	Douala, Cameroon
Type of facility found in	Private pharmacy	Private pharmacy	Informal vendor	Private pharmacy	Informal vendor
Date of discovery	April 3, 2020	March 31, 2020	April 4, 2020	April 4, 2020	March 31, 2020
Labeling inconsistencies					
Spelling errors	**+**	−	**+**	−	**+**
Invalid NAFDAC registration number	**+**	**+**	−	**+**	**+**
Same batch number for different products	**+**	**+**	−	**+**	**+**

DR = Democratic Republic; NAFDAC = National Agency for Food and Drug Administration and Control, Nigeria. NAFDAC registration numbers were checked using the NAFDAC Registered Products Database available at www.nafdac.gov.ng/our-services/registered-products/.

Thin layer chromatography readily showed the presence of CQ in the reference solutions, visible both under UV light and in subsequent detection with iodine vapor. By contrast, CQ was not detectable in four of the investigated samples. The fifth sample showed a spot of CQ, but the compound was apparently present only in a low amount (Figure 1, sample I). For samples II and III (Figure 1), TLC analysis with UV detection showed the presence of further, undeclared compounds with a higher retention factor than CQ. The undeclared compound in sample II was also detectable by iodine staining, but the compound in sample III was not (Figure 1), indicating that these two compounds were chemically different.

These observations were confirmed at Tübingen University by high-performance liquid chromatography (HPLC) according to the U.S. Pharmacopeia.^13^ As shown in Figure 2, no CQ was detected in four of the samples. By contrast, in sample I, CQ was present in an amount corresponding to 21.7 mg CQ phosphate, that is, only 21.7% of the amount stated on the label. Samples II and V showed an unknown compound with a retention time of 4.7 minutes in HPLC, and samples III, IV, and V showed a further unknown compound with a retention time of 4.5 minutes.

Liquid chromatography (LC) coupled with high-resolution mass spectrometry (HR-MS) showed that the two unknown compounds had exact molecular masses of 152.0709 and 172.0719, consistent with the masses of paracetamol and of metronidazole, respectively. Their identity was confirmed in comparison with authentic reference compounds of paracetamol and of metronidazole, showing identical retention times, molecular masses, and mass spectrometric fragmentation as the references (Supplemental Table S4, Supplemental Figures S2 and S3, Supplemental Information). The quantities of these compounds were determined as 35.7 mg paracetamol per tablet for sample II and as 126.5 mg metronidazole per tablet for sample III. Samples IV and V were found to contain smaller amounts of metronidazole, that is, 14.1 mg and 14.6 mg per tablet, respectively. Sample V additionally contained traces of paracetamol (1.6 mg per tablet).

The labeling of the five samples showed mistakes and spelling errors (Table 1), suggesting that they were produced not by established manufacturers but by criminals. The stated manufacturer of sample III, Dawa Limited, Kenya, was contacted by the local partners in the DR Congo and confirmed that this sample had not been produced by them. Samples I, II, IV, and V were stated to be produced by “Jiangsu Pharmaceutical Inc., China,” but no company with that name, or with the address stated on the labels, could be identified on the internet.

Notably, while this report was in preparation, Cameroon customs authorities reported the seizure of 210 cartons of falsified CQ tablets.^14^

The low amount of CQ in sample I is likely to reflect the attempt by the criminal producers to save costs in the purchase of the active pharmaceutical ingredient. The inclusion of paracetamol, as in sample II, has been reported previously in a falsified medicine from Cameroon, also identified by members of EPN.^10,15^ Both in sample II and in that previous case, the amount of paracetamol was too low to achieve a relevant therapeutic effect. Metronidazole is very bitter and was included in samples III, IV, and V probably to mimic the bitter taste of CQ. The antibacterial and antiprotozoal compound metronidazole is usually formulated in tablets of 200–500 mg each. Therefore, samples III, IV, and V contain a subtherapeutic dose, which may contribute to the emergence of antimicrobial resistance. The additional presence of traces of paracetamol in sample V may represent a contamination from a prior production batch, reflecting poor manufacturing standards.

The absence of CQ in four of the five investigated samples, the subtherapeutic amount of CQ in the fifth sample, and the presence of undeclared active pharmaceutical ingredients in four of these samples represent serious health risks for the patients in Cameroon and the DR Congo. The authorities in Cameroon and the DR Congo, and the WHO Rapid Alert System were informed about these findings.

Such products may furthermore cause financial hardships to the patients: sample III was sold in the DR Congo for US$200 for a package of 1,000 tablets, that is, 15 times more expensive than the international procurement price.^16^ In Cameroon, the EPN partner organization even reported the occurrence of a further package of 100 CQ tablets with a stated price of 250,000 CFA, that is, US$413 (Supplemental Figure S1, Supplemental Information).

The occurrence of such falsified CQ samples at this time of the COVID-19 pandemic also has wider implications. For any medicine or vaccine which may be reported to be effective against this disease, a frantic demand is to be expected, resulting in a serious danger of the appearance of falsified medicines. Low- and middle-income countries (LMICs) will be especially vulnerable: with their constrained access to essential medicines, their often weak technical capacity for medicine quality assurance and control, and their challenges in the maintenance of appropriate standards of governance in healthcare facilities and national medicines regulatory authorities, they show exactly those conditions which the WHO has identified as favoring the occurrence of substandard and falsified medicines.^17^ Because of the recent disruption of the production and supply chains in India and China, which are the most important producer countries of generic medicines for LMICs, this problem will not remain restricted to medicines for the treatment and prevention of COVID-19 but encompass many types of medicines.

The rapid installation of simple, inexpensive screening technologies which can detect substandard and falsified medicines, such as TLC or near infrared or Raman spectroscopy,^8,9,18^ may represent an important part of the response to the COVID-19 pandemic in LMICs. The data displayed in Figure 1 are a good example for the possibilities and limitations of the GPHF Minilab^11^ in the identification of falsified medicines in future screening programs.

## Supplemental tables and figures

Supplemental materials
