# Supplementary material for: Identification of Falsified Chloroquine Tablets in Africa at the Time of the COVID-19 Pandemic
Source: Am J Trop Med Hyg. 2020 May 12;103(1):73–6. doi: 10.4269/ajtmh.20-0363 (PMC7263564; doi:10.4269/ajtmh.20-0363)
Supplement: Supplementary file 1 [file tpmd200363.SD1.pdf]

Supplemental Information for

The identification of falsified chloroquine tablets in Africa at the time of the COVID-19 pandemic

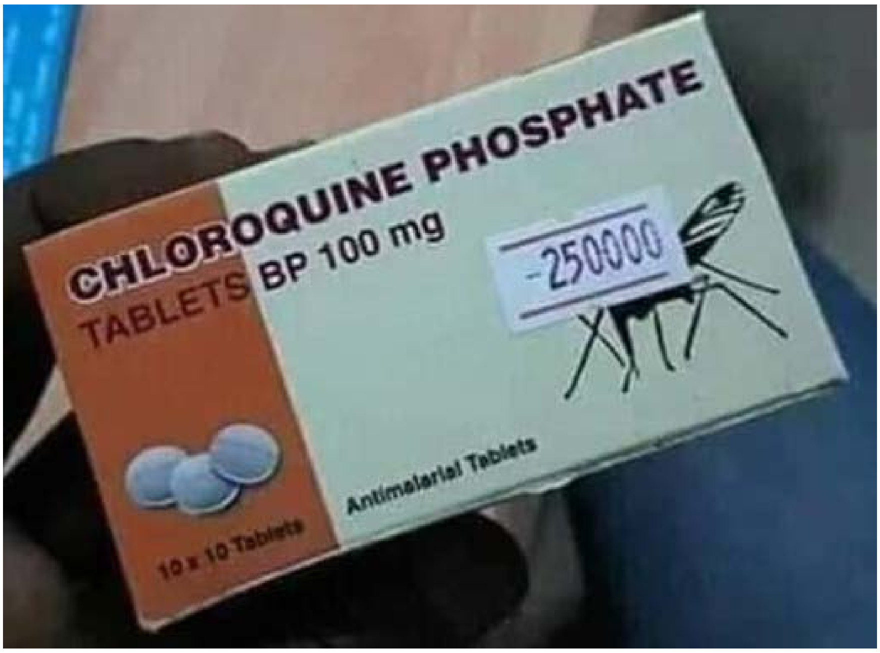

Figure S1: *Package of 100 chloroquine phosphate tablets 100 mg found in Yaoundé, Cameroon, on April 9, 2020, with a stated price of 250,000 CFA, i.e. 414 US $ (Photo: © F. Nyaah). Due to the exorbitant price, this sample was not purchased and not analyzed.*

Methods of thin-layer chromatography and quantitative HPLC analysis

Method	Chloroquine phosphate and sulfate (GPHF Minilab Manual 2020)[1]
Stationary phase	Merck TLC aluminium plates pre-coated with silica gel 60 F_{254} , 5x10 cm
Mobile phase	Ethyl acetate/methanol/25% aqueous ammonia solution 1:4:0.1 (v/v)
Applied volume of sample and standard	2 µl
Detection	1) UV light, 254 nm;<br>2) Exposure to iodine vapor and visual evaluation in daylight
Standards	Chloroquine phosphate in water, 2.5 and 2.0 mg/ml
Sample preparation	Tablets with a declared content of 100 mg chloroquine phosphate: one tablet was finely ground with a pestle and suspended in 20 ml of water.<br>(Tablets with a declared content 250 mg chloroquine phosphate: one tablet was finely ground with a pestle and suspended in 50 ml of water.)<br>After three minutes of shaking, the solution was allowed to sit for additional five minutes. 2 ml of the supernatant were removed and diluted with 2 ml of water.

Table S1: *Method for thin-layer chromatography*

Method	Chloroquine Phosphate Tablets (USP 42 monograph, 2019)[2]
Instrument	HPLC (Agilent 1100 Series)
Column/stationary phase	Reprospher 100 C18, 250 x 4 mm, 5µm (Dr. Maisch GmbH, Ammerbuch, Germany)
Mobile phase	Methanol/aqueous buffer 22:78 (v/v)<br>(aqueous buffer contained 6.8 g monobasic potassium phosphate and 1 ml perchloric acid per liter water; pH 2.5)
Flow rate	1.2 ml/min
Oven temperature	30 °C
Injection volume	10 µl
Detector	UV, 224 nm
Standard	0.15 mg/ml chloroquine phosphate Pharmaceutical Secondary Standard (Sigma-Aldrich LOT #LRAB3715) in water.
Sample preparation	One tablet was finely ground in a mortar. An aliquot of approx. 100 mg was weighed into a 100 ml volumetric flask. 50 ml of water were added. The flask was sonicated for 15 minutes and then filled up with water to 100 ml. For each sample, two independent experiments were carried out.

Table S2: *Method for quantitative HPLC analysis of chloroquine and paracetamol*

Method	Metronidazole Tablets (USP 42 monograph 2019)[3]
Instrument	HPLC (Agilent 1100 Series)
Column/stationary phase	Reprospher 100 C8, 150 x 4.6 mm, 5µm (Dr. Maisch GmbH, Ammerbuch, Germany)
Mobile phase	Methanol/water 20:80 (v/v)
Flow rate	1.0 ml/min
Oven temperature	30 °C
Injection volume	5 µl
Detector	UV, 254 nm
Standard	0.56 mg/ml Metronidazole Analytical Standard (Sigma-Aldrich LOT #MKBZ3056V) in methanol/water 20:80 (v/v).
Sample preparation	Three tablets were finely ground in a mortar. An aliquot of approx. 100 mg was weighed into a 100 ml volumetric flask. 50 ml of methanol were added. The flask was sonicated for 10 minutes and then filled up to 100 ml with mobile phase. For each sample, two aliquots were weighted and analyzed.

Table S3: *Method for quantitative HPLC analysis of metronidazole*

High resolution liquid chromatography-mass spectrometry

HR-HPLC/MS(/MS) was carried out using a Thermofisher UltiMate 3000 HPLC with a Phenomenex Luna 3µm Polar C18 100 Å column 150 x 2 mm, column temperature 30°C. Eluent A: 0.1% formic acid in water; eluent B: 0.1% formic acid in methanol. Gradient 5-100% B over 20 min followed by 100% B isocratic for 10 min; flow rate 0.3 ml/min. UV detection with a diode array detector. HR mass spectrometry: ESI-TOF Bruker MaXis 4G. The sample solutions were investigated in comparison to authentic paracetamol and metronidazole reference in H_2O/methanol 2:1. In samples III, IV and V, peaks at 5.0 min (metronidazole) were detected. In samples II and V, peaks at 5.4 min (paracetamol) were detected. UV spectra of the samples and the respective references were identical. The molecular ion of the respective samples showed the same exact mass as the molecular ion from paracetamol and/or metronidazole (Table S4). These were consistent with the molecular formula $C_8H_9NO_2$ of paracetamol and $C_6H_9N_3O_3$ of metronidazole.

Sample	Retention time	$[M+H]^+_{theoretical}$	$[M+H]^+_{measured}$	relative mass accuracy
Metronidazole reference	5.0 min	172.0717	172.0719	1.3 ppm
Paracetamol reference	5.4 min	152.0706	152.0709	2.0 ppm
Sample II	5.4 min	152.0706	152.0709	1.8 ppm
Sample III	5.0 min	172.0717	172.0721	2.3 ppm
Sample IV	5.0 min	172.0717	172.0720	1.9 ppm
Sample V	5.0 min	172.0717	172.0718	1.0 ppm
	5.4 min	152.0706	152.0708	1.2 ppm

Table S4: *Retention times, and theoretical and measured exact masses, for the investigated samples and for metronidazole and paracetamol reference substances. HPLC conditions for HPLC-MS are different from those for quantitative analysis according to USP, therefore retention times are different from those shown in Figure 2.*

MS/MS analysis showed the presence of the characteristic fragments of metronidazole (Fig. S2) in samples III, IV and V, and the presence of the characteristic fragments of paracetamol (Fig. S3) in samples II and V. The observed fragmentation of the samples and the respective references were identical.

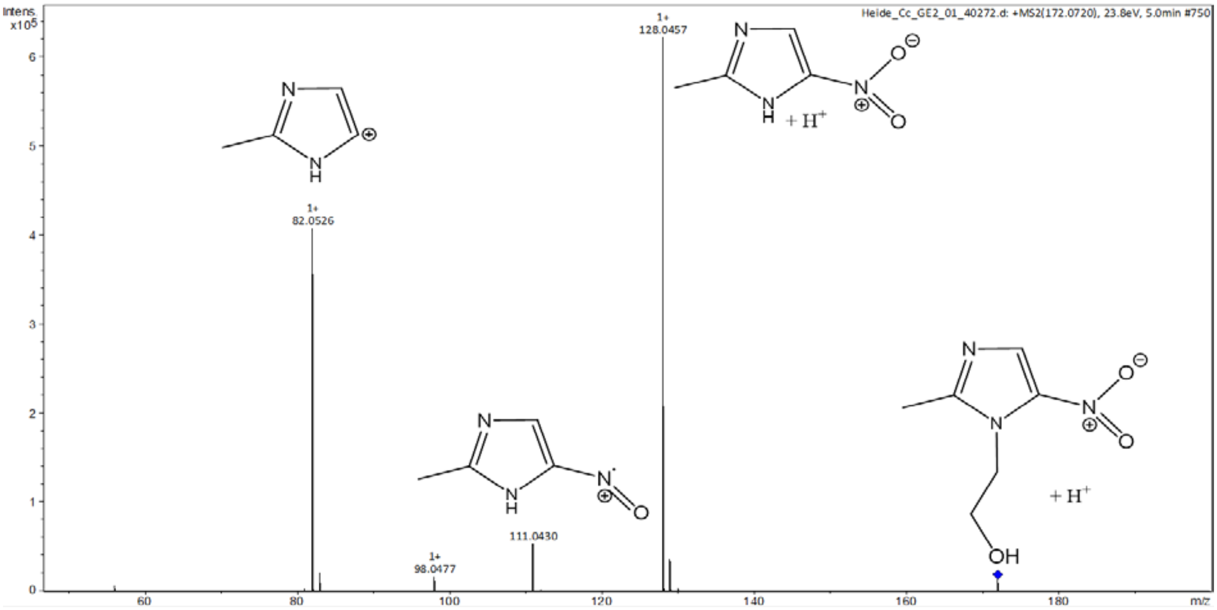

Figure S2: *MS/MS fragmentation of metronidazole in sample IV.*

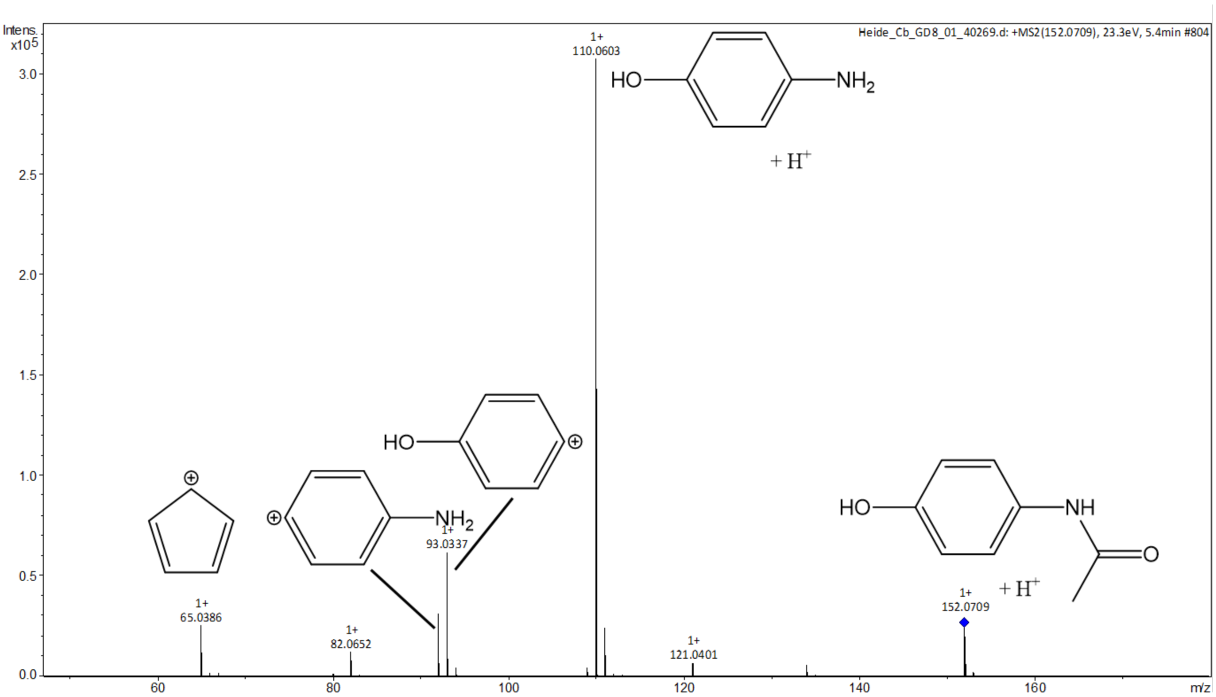

Figure S3: *MS/MS fragmentation of paracetamol in sample II.*

References:

1. Jähnke WO, Dwornik K, 2020. Manual accompanying the GPHF Minilab™. Physical testing and thin-layer chromatography. Giessen, Germany: Global Pharma Health Fund.
2. United States Pharmacopeia 42 NF 37, 2019. Monograph: Chloroquine Phosphate Tablets. USP, Rockville, USA.
3. United States Pharmacopeia 42 NF 37, 2019. Monograph: Metronidazole Tablets. USP, Rockville, USA.
